# Effect of Maternal Diet and Medium Chain Fatty Acids Supplementation for Piglets on Their Digestive Tract Development, Structure, and Chyme Acidity as Well as Performance and Health Status

**DOI:** 10.3390/ani10050834

**Published:** 2020-05-11

**Authors:** Małgorzata Świątkiewicz, Ewa Hanczakowska, Krzysztof Okoń, Piotr Kowalczyk, Eugeniusz R. Grela

**Affiliations:** 1 Department of Animal Nutrition and Feed Science, National Research Institute of Animal Production, Krakowska 1, 32-083 Balice, Poland; ewa.hanczakowska@izoo.krakow.pl; 2 Department of Pathomorphology, Medical College, Jagiellonian University, Grzegórzecka 16, 31-531 Krakow, Poland; k.okon@cm-uj.krakow.pl; 3 Department of Biophysics, Faculty of Environmental Biology, University of Life Sciences in Lublin, Akademicka13, 20-950 Lublin, Poland; lekwetpiotrkowalczyk@gmail.com; 4 Auxilium Veterinary Clinic, Królewska 64, 20-950 Milanówek, Poland; 5 Institute of Animal Nutrition and Bromatology, Faculty of Animal Sciences and Bioeconomy, University of Life Sciences in Lublin, Akademicka 12, 20-950 Lublin, Poland; eugeniusz.grela@umcs.lublin.pl

**Keywords:** coconut oil, medium chain fatty acids, piglets, sow milk fatty acids, intestine structure, intestine chyme acidity, immunoglobulins, biochemical blood indices, growth performance

## Abstract

**Simple Summary:**

Weaning is the most critical period of piglet rearing. During this time, pigs have not yet fully developed their intestinal tract and immune system; therefore, they are an easy target for pathogenic microorganisms that cause gastrointestinal diseases. In the last decade, several nutritional factors were studied to prevent gastrointestinal disorders in piglets. The present study aimed to evaluate the effect of oils for sows during late pregnancy and lactation on offspring performance. In addition, the study determined the effect of caprylic acid or medium-chain triglyceride oil in piglets’ feed on their intestinal structure development, fatty acids content of chyme, productive performance, and health status. Summarizing, the study showed that coconut oil fed to pregnant and lactating sows can markedly reduce the mortality of piglets during the weaning period and that caprylic acid and medium-chain fatty acid oil can be a good feed supplement in weaned piglet feed. The nutritional factors tested in the present study could be used in the diet of sows and piglets to improve the health of piglets and thus the efficiency of pig production.

**Abstract:**

The objective of the present study was to evaluate the effect of oils for sows during late pregnancy and lactation on offspring performance. In addition, the effect of caprylic acid (C8) or medium-chain triglyceride oil (MCT) in piglets’ feed on their gut development, performance, and health status was determined. The experiment was conducted on 24 sows allocated to two treatments: diet with rapeseed oil or with coconut oil. Newborn piglets were randomly allocated to three treatments: feed with no supplement or supplemented with 0.3% MCT or with 0.3% C8. The results showed that both oils had no effect on sow reproductive rates; however, fatty acid patterns of milk differed significantly and the number of lost piglets was lower in sow fed with coconut oil. Both caprylic and MCT oil significantly improved piglet performance and villus height. These additives did not change triacylglycerol content in blood, but C8 lowered total cholesterol and MCT increased IgG content. It can be concluded that coconut oil fed to pregnant and lactating sows can markedly reduce the mortality of piglets and that caprylic acid and medium-chain fatty acid oil can be a good supplement in weaned piglet feed.

## 1. Introduction

Supplementation of the diet of sows during late pregnancy and lactation is one of the less popular ways to positively affect the health status of offspring and/or the productive indices of sows.

Our earlier experiment showed that the intensive feeding sows during early pregnancy (time period 25–55 days) can have a positive effect on the body weight of born piglets and increase the amount of IIB type muscle fibers as well as their diameter in *longissimus* m. [1]. In the experiment of Lavery et al. [2], neither the energy concentration nor the oil type fed to lactating sows affected the sow body condition and piglets’ growth performance. However, there was a tendency for salmon oil to decrease piglet mortality as well as increase sow milk yield and change the milk fatty acids profile. Dietary supplementation of sows with polyunsaturated fatty acids n-3 (PUFA n-3) affects the colostrum composition to the greatest extent by increasing the content of fat and protein, while medium-chain fatty acids (MCFA) additive shortens the weaning-to-estrus interval of sows [3]. However, with regard to offspring, these authors observed more positive effect on intestinal health and lower mortality of suckling piglets when dietary sodium butyrate was used for pregnant sows compared to the results observed for dietary MCFA or PUFA n-3. The experiment of Vodolazska and Lauridsen [4] confirmed that the maternal dietary fatty acid profile of oil influence the fatty acid profile in sows’ colostrum and mature milk, blood plasma, and plasma of their progeny.

Weaning is the first critical period in the life of piglets. During this time, they have not yet fully developed their intestinal tract and immune system [5]. Therefore, they are an easy target for pathogenic microorganisms causing gastrointestinal diseases [6]. They also have to adapt to new stressful conditions, which results in reduction of feed consumption, temporary malnutrition, and growth retardation [7]. Antibiotic growth promoters were used to prevent these issues, but in recent years, they have been banned by the European Union [8]. The reduction of villus length and increased crypt depth and enzyme activity are often observed during the first 3–5 days post-weaning, which predispose the piglets to gastrointestinal disorders and consequently lead to much slower growth rate during the post-weaning period [9]. It is known that ileum, especially the Peyer’s patches located in its terminal region, plays a crucial role in targeting antigens and act as a first line of blockage of pathogens in the small intestine [10]. Various approaches have been proposed to improve the ileum health status and prevent gastrointestinal disorders in piglets. The most popular solutions include herbal extracts, pre-and probiotics, and acidifiers, which are mainly known for gut development, antibacterial resistance, disease prevention, and growth promotion effects [11,12,13]. These include MCFA, which could be considered as substitutes for antibiotics [14]. These fatty acids are caproic (C6:0), caprylic (C8:0), capric (C10:0), and lauric acids (C12:0). As a result of their relatively low molecular weight and size, MCFA are more soluble in water and biological liquids than in long-chain fatty acids [15]. They have also strong antibacterial activity [16] and can improve post-weaning gut development [17]. As intestinal epithelium cells are the main site of nutrient absorption, provision of such easily absorbable nutrients should improve their structure and function [18]. Positive changes in gut structure (greater villus height) may result in improved performance of piglets, as reported by Hanczakowska et al. [19]. Li et al. [20] showed that piglets fed MCFA supplement grew faster than controls receiving soybean oil during the first two weeks of the experiment. Later, there was no difference in weight gain of piglets in the experimental and control groups. This early improvement of performance could be due to traits of MCFA such as better solubility and shorter path of absorption.

The activity of fatty acids is not the same. In our earlier experiment on piglets that compared the effect of dietary supplements of caprylic and capric acids [19], we found that piglets receiving fatty acids with a shorter chain length (caprylic) grew faster than those receiving both these fatty acids or capric acid alone. However, in a later experiment [21], capric acid yielded better results than caprylic acid. Significant differences were also found in the utilization of MCFA triacylglycerols [22]. When they were given as separate substrates, lipase activity decreased progressively as fatty acid chain length increased from C4 to C10. When they were given as combined substrates, tri-C6 was hydrolyzed most rapidly, followed by C4:0, C8:0, and C10:0. The hydrolysis of C6:0 was seven times greater than that of C8:0.

Present study aimed to evaluate the possibility of the synergistic effects of various oils given to sows and a mixture of different MCFA as a supplement in feed for their offspring on the performance and health status of piglets in comparison to the effect of caprylic acid alone.

## 2. Materials and Methods

### 2.1. Sows, Piglets, Diets, and Experimental Scheme

All procedures used in this experiment were approved by the Second Local Cracow Ethics Committee for Experiments on Animals of the Polish Academy of Sciences (Resolution No 1104/2014, of 24 July 2014).

In the experiment, 24 sows of the Polish Landrace mated with the Duroc × Pietrain boar were used. Sows were divided into 2 treatments, each contained 12 sows. During late pregnancy (from 100 d after mating) and lactation, both treatments were fed a standard feed mixture but differed in fodder fat: group I received rapeseed oil (RO), group II received coconut oil (CO) (Table 1). Sows were kept individually and fed a feed at the amount of 3.2 kg per sow during late gestation. During lactation, feeding rate were dependent on the litter size. Each sow was weighed at the 100th d of gestation, at farrowing, and weaning. Milk samples from each sow were collected at 14th d of lactation and frozen at −18 °C for further analysis of fatty acid content.

After farrowing, piglets from each group of sows were randomly assigned to 3 groups, according to the scheme: 4 litters were assigned to group I—feed with no supplement (NS), 4 litters were assigned to group II—feed supplemented with 0.3% medium chain triglyceride (MCT), 4 litters were assigned to group III—feed supplemented with 0.3% caprylic acid (C8). Examined MCT oil contained 0.01% of caproic acid, 60.69% of caprylic acid, 38.78% of capric acid, and 0.51% of lauric acid, (Olimp Laboratories Sp. z o.o., Dębica, Poland). Each group of piglets contained 12 litters, group I—100 piglets, group II—90 piglets, group 3—91 piglets. Piglets were kept in group pens, 1 litter in each pen. Piglets were fed ad libitum since the 7th day of age, and the feed mixtures in all groups were isoenergetic and isonutrigenous and covered the piglets’ requirements for nutrients, but differed in supplement (no supplement, MCT, or C8). The composition of feeds for the piglets is given in Table 2.

The piglets were weighed individually on the 1st, 7th, 28th, 56th, and 84th day of life. They were weaned at 28 days of age and reared in litters. Feed consumption by each litter was measured daily and feed utilization as well as average daily weight gains were calculated. The number of born, dead, and culled piglets was monitored. On the 21st d of life, blood samples were taken from the jugular vein of randomly chosen 6 piglets from each piglets’ group (NS, MCT, and C8). On the 60th d of life, 6 piglets from each piglets’ group (NS, MCT, and C8) were sacrificed in order to take the samples of blood and to prepare the digestive tract. The acidity of chyme was measured at the consecutive main parts of the alimentary tract. The acidity of the intestines content was measured with a pH-meter CP—411 (Elmetron, Zabrze, Poland) equipped with a Metron 12—01 electrode (Metron, Toruń, Poland). The samples of digesta from the jejunum and cecum were taken to analyze the short chain fatty acids content. Samples of duodenum and jejunum from their middle section were taken for histological analysis of epithelium structure. Content of intestines was removed, and the length and weight of elementary tract segments were examined.

### 2.2. Blood Analyses

In blood serum taken from piglets on the 21st and 60th d of life, IgA, IgM, IgG antibodies, and C-Reactive Protein (CRP) were analyzed using turbidimetry kits (Biosystem, Barcelona, Spain) with an automatic analyzer (DS261) according to the manufacturer’s instructions. Daily calibrations were carried out for all measurements made during the study.

Blood serum taken on the 60th d of life was analyzed for triacylglycerols (TG), cholesterol (CHOL), high-density lipoprotein fraction (HDL), and free fatty acids (FFA). Alkaline phosphatase (ALP), lactate dehydrogenase (LDH), aspartate aminotransferase (AST), and alanine aminotransferase (ALT) were also determined. The biochemical analysis of the aforementioned parameters was made using the colorimetric methods and monotests of Cormay and BioMaxima, a Metrolab biochemical analyzer (Metrolab, Buenos Aires, Argentina), and a Cary 50 spectrophotometer (Varian, part of Agilent Technologies, Santa Clara, CA, USA). The low-density lipoprotein fraction (LDL) was calculated from the Friedewald et al. [24] equation.

### 2.3. Chemical Analyses

Gross composition of feeds was estimated according to AOAC [25] methods. Samples for fatty acid analysis were extracted with the chloroform/methanol mixture (2:1 v/v) as described by Folch et al. [26]. Fatty acids were saponified (0.5 N NaOH in methanol) and next esterified with boron trifluoride/methanol [27]. Fatty acid profiles of feed, MCT oil and sow milk were determined using a CP-Wax 58 capillary column (Varian BV, Middelburg, the Netherlands) (25 m, 0.53 mm, df—1 µ, carrier gas—helium, 6 mL/min), with a column oven temperature program from 90 to 200 °C, using a Varian 3400 gas chromatograph (Varian Instruments Inc., Walnut Creek, CA, USA) equipped with a Varian 8200 CX Autosampler (200 °C), FID detector (260 °C), and Star Chromatography Workstation Software (Varian Instruments Inc., Walnut Creek, CA, USA).

Volatile fatty acids content of jejunum and cecum chyme were analyzed by gas chromatography after centrifugation of aqueous filtrates with 24% meta-phosphoric acid. They were separated on a CP-Wax 58 column (Varian Instruments Inc., Walnut Creek, CA, USA) (25 m, 0.53 mm, 1 m, carrier gas—helium, 6 mL/min), with a column oven temperature program from 90 to 200 °C, using a Varian 3400 gas chromatograph (Varian Instruments Inc., Walnut Creek, CA, USA) equipped with a Varian 8200 CX autosampler (200 °C), FID detector (260 °C), and Star Chromatography Workstation Software (Varian Instruments Inc., Walnut Creek, CA, USA). All analyses were performed in duplicate and mean values are given.

### 2.4. Histological Analysis

Samples of the duodenum and jejunum were taken in order to measure the length and width of the villi, as well as the crypts depth. The fragments were cut out across the entire intestinal wall, from the middle section of each duodenum and jejunum. The collected material was unbuttoned on pieces of polystyrene and fixed in a 10% solution of buffered formalin. After fixation, each fragment of the intestinal wall was gently cut, preparing 4 sections (preparations) per fragment. Preparations were routinely performed using a tissue processor (Shandon Inc., UK). Next, paraffin blocks were prepared, from which 4 µm thick slices were made, placed on microscope slides and stained with the hematoxylin-eosin method. Images acquisition was carried out using a Zeiss Axioscop microscope (Zeiss GmbH, Germany) with a Plan-NEOFLUAR 2.5 × lens and ZVS-47DE CCD camera (Optronics Inc. USA), connected by an RGB line with a GraBIT PCI card (Soft Imaging System GmbH, Germany) installed on a standard PC. The system has been scaled using a microscope slide with a graduation of 10 µm. The obtained images had a resolution of 768 × 576 points, which corresponded to the size of the field of view 2573.83 × 1929.53 mm. The acquisition, image processing and measurement software worked under the control of the Windows NT 4.0 operating system and consisted of the image analysis system AnalySIS 3.0 pro (Soft Imaging System GmbH, Germany) and application software prepared in the language Imaging C (ANSI C) especially for this purpose. Each preparation was viewed under a microscope looking for all well-preserved villi and crypts. The well-preserved villi were those that were straight, with visible epithelial covering and vessels in the stroma. The well-preserved intestinal crypts were those whose lumen as well as the lining of the epithelium was well preserved over the entire length, the course of light was straight and they were visible from the estuary to the muscularis of the mucosa.

### 2.5. Statistical Analysis

Statistical analysis of the fatty acids content in milk and sows’ reproductive rates was performed using one-way analysis of variance (ANOVA) with type of oil as the factor. The remaining results were calculated by two-way ANOVA, using a 2 × 3 factorial arrangement with type of oil and supplemental medium as the factors. The interaction between type of oil and supplemental medium was added to the model. The ANOVA assumptions were tested using the Shapiro–Wilk test for normality and Levene’s test for homogeneity of variances. Comparisons of means were made by Tukey’s multiple range test at *p* ≤ 0.05 levels of significance using the Statistica 10 package [28].

## 3. Results

### 3.1. The Effect of Oils in Maternal Diet on the Offspring and the Fatty Acids Profile of Milk

The type of oil added to the sow diet during late pregnancy did not affected their reproductive indices, including the body weight of newborn piglets, but the piglet losses were lower in group fed with coconut oil (Table 3).

The type of oil in the sow lactation diet influenced the fatty acid profile of the sow’s milk (Table 4). In general, the milk of sows fed with coconut oil contained more fatty acids, especially lauric (C12:0) and myristic (C14:0) acids (*p* < 0.01). The milk of sows fed with rapeseed oil contained more long carbon chain fatty acids, especially linoleic, linolenic n-6, and arachidic (*p* < 0.01). This milk also contained more unsaturated fatty acids PUFA, i.e., eicosapentanoic EPA (*p* = 0.04) and docosahexaenoic acids DHA (*p* = 0.02).

### 3.2. Growth Performance of Piglets

Piglets from sows receiving coconut oil grew faster than those from sows receiving rapeseed oil (Table 5), especially during the last rearing period of between 56 and 84 days of age (*p* < 0.05). In the subgroups, the lowest weight gains were mainly observed in the control piglets, while the highest weight gains were found in piglets receiving caprylic acid. Piglets receiving fatty acid supplementation (MCT or C8) grew faster than controls (*p* < 0.05). During suckling period (1–28 days), piglets receiving MCT or C8 grew faster (9.5% or 8.6%, respectively) compared to controls (*p* < 0.05). From weaning to the end of the experiment, piglets receiving caprylic acid grew faster than control animals (17.4%) and fed MCT oil (8.6%) (*p* < 0.05). During the whole experiment (1–84 day), body weight gains of piglets were 278, 301, and 320 g in the control, MCT, and C8 acid groups, respectively. The difference between the caprylic acid and control group (about 15%), and between the MCT and control group (about 8%) were significant. Significant interaction between body weight gains of piglets from sows fed different oils and these receiving different supplements was found only in the first period i.e., between 1 and 28 day of age. The type of oil and fatty acid supplements had no significant effect on feed consumption or utilization (data not shown). After weaning period (28–56 day of age) piglets fed with MCT or C8 utilized feed better than control ones (4.6% or 10.3%, respectively), but the differences were not statistically significant.

### 3.3. Acidity of Chyme and Volatile Fatty Acids Content

Both supplements used had only a limited effect on the acidity of chyme in the piglet digestive tract (Table 6). Changes were limited to the duodenum in which coconut oil given to sows, (and also MCT oil and caprylic acid (*p* < 0.01) lowered pH. There were also only small differences in short chain fatty acids content in jejunum and cecum chime (Table 7). Examined MCT oil increased (*p* = 0.02) content of isovaleric acid in jejunum and lowered (*p* < 0.01) butyric and valeric acids content in the cecum. These differences, together with the apparent lower content of acetic and propionic acids resulted in a lower total content of volatile fatty acid (VFA) (*p* = 0.03) in the cecum of piglets receiving MCT oil.

### 3.4. Digestive Tract Development and Histological Structure

The type of oil given to sows had no effect on the piglets’ digestive tract mass nor on the estimated mass of internal organs (Table 8). Piglets fed with MCT oil had the heaviest total intestinal mass (*p* = 0.02), which was the result of a relatively higher mass (*p* < 0.01) of the duodenum and cecum. All parts of the intestinal tract of piglets receiving caprylic acid (C8) were shorter than those the control group (NS, *p* < 0.01), and apart from the colon, were also shorter than those fed MCT oil. As a result of these differences, the total intestinal length of piglets receiving caprylic acid (C8) was shorter (about 16%) than in control piglets (*p* < 0.01), and shorter (about 14%) than those fed with oil MCT (*p* < 0.01). The livers (*p* = 0.03) of piglets receiving MCT as well as their spleens (*p* < 0.01) had higher mass than in control animals (NS).

The type of oil given to sows had no effect on villi height in the duodenum and jejunum (Table 9). The villi height in the duodenum of piglets fed with caprylic acid (C8) was higher (*p* = 0.04) than in the control group by 26.2%. The supplementation with MCT oil resulted in a 6.2% increase in villi height. In the jejunum piglets fed with MCT oil villi were wider than in the control group (*p* < 0.01). 

### 3.5. Biochemical Blood Indices and Immunoglobins Content in Piglets

The type of oil fed to sows had a distinct effect on cholesterol content in piglet blood on the 60 d of their life (Table 10). Piglets from sows treated during late pregnancy and lactation with rapeseed oil had (*p* < 0.01) lower total cholesterol, as well as both of its fractions. On the other hand, they had higher levels of triacylglycerols (TG), free fatty acids (FFA), and aspartate aminotransferase (AST) in the blood (*p* < 0.01). MCT oil supplementation in piglet feed had no effect on cholesterol content in the blood, but caprylic acid lowered it (*p* < 0.01). Neither MCT oil nor caprylic acid changed the TG or FFA content in the piglets’ blood. Piglets receiving caprylic acid had lower (*p* < 0.01) level of alanine aminotransferase (ALT) in the blood than those fed a control diet or MCT oil. An interaction was found between the type of oil and the type of supplement in the case of total and LDL cholesterol, as well as alkaline phosphatase (ALP) and ALT enzymes (*p* < 0.01).

The content of IgM and IgG on the 21 d of a piglet’s life depended on the type of oil in the sow’s feed. Greater levels of IgM and IgG were found in piglets from sows fed with coconut oil (*p* < 0.01). There was no such effect on the 60 d of life (Table 11). Caprylic acid lowered all immunoglobulins in the first period of the experiment (21 days of age), in the case of IgM and IgG this decrease was statistically significant (*p* < 0.05). MCT oil had no effect on immunoglobulin levels in this phase. Later (60 days of age) caprylic acid did not cause any difference, but MCT oil increased IgG levels in the blood (*p* < 0.01). C-reactive protein (CRP) levels were similar in all piglets at 21 and 60 days of age (Table 11).

## 4. Discussion

### 4.1. Effect of Oils in Maternal Feeding on the Sows Reproductive Performance and Health Status of the Offspring

Coconut oil is a rich, natural source of MCFA [29]. MCFA, which are a good feed supplement for piglets, are not frequently used in sow feeding; however, Chen et al. [3] reported that MCFA fed to sows during late pregnancy and lactation shortened the weaning-to-estrus interval, reduced the incidence of diarrhea in suckling piglets, and increased the protein, IgA, IgG, and IgM content in colostrum. Supplementation with coconut oil increased the content of MCFA, mainly lauric acid, in milk, but there was no significant difference in the number of piglets born or in their mean body weight. On the other hand, the number of lost piglets was apparently lower in the group fed with coconut oil. Unfortunately, MCFA present in coconut oil significantly lowered eicosapentaenoic (EPA) and docosahexaenoic (DHA) acid content in milk. Both these fatty acids are important from the physiological point of view [30]. A similar effect of MCT was found in human blood by Fergusson et al. [31]. Jean and Chiang [32] fed sows with MCT and found that it could be an effective means of increasing the survival of neonatal pigs. According to the authors, this improvement might be due to an enhancement of body’s glycogen stores and maturity of pigs at birth. Azain [33] showed that MCT supplementation increased the content of MCFA in milk (less than 5%), but improvement in piglet survival was observed mainly in animals weighing less than 900 g at birth. On the other hand, Chen et al. [34] found a greater concentration of C18:3 fatty acids in the milk of sows fed linseed oil than in the milk of those receiving soybean oil. Linseed oil also improved the weaning weight of piglets and their average daily weight gain. Similarly, the milk profile was increased in PUFA n-3 when sow were fed with diet containing the hemp seed oil during the late pregnancy and lactation, and piglets were able to convert these fatty acids obtained via the sow milk intake to C20:5 n-3 and C22:5 n-3. In addition, the oil type (hemp seed oil) in maternal diet positively increased the number of piglets born and decreased their mortality as well as the oil type affected the concentration of immunoglobins in blood of piglets [4].

The significantly better growth rate of piglets descending from sows fed with coconut oil is difficult to explain. Perhaps, they were better accustomed to MCFA (until the 28th day of their life, they could consume coconut oil while remaining with sows), and later, they could utilize feed containing these acids more efficiently. These piglets also utilized feed slightly (not significantly) better than those from the rapeseed oil group. Chen et al. [34] compared soybean and linseed oils and indicated that the type of oil in the sow’s diet affected the body weight gain of piglets.

The greater cholesterol level in the blood of piglets receiving milk from sows fed with coconut oil was probably due to the greater content of saturated fatty acids, especially lauric and myristic acids, in this milk. According to Mensink et al. [35], these fatty acids (and also palmitic acid) are more hypercholesterolemic than saturated fatty acids with a shorter carbon chain. Lower free fatty acids content in the blood of piglets receiving milk from sows fed coconut oil could be the result of a greater content of MCFA in this milk. As a result of their low molecular weight and size, MCFA are highly soluble in water and biological liquids, and they are absorbed directly into portal circulation and transported to the liver for rapid oxidation [36]. The type of oil fed to sows had an apparent effect on the immune system of their progeny at the early period of life. This is in accordance with the results of Chen et al. [34], who found increased levels of IgG and IgA after supplementation of maternal feed with linseed oil. This effect disappeared before the 60th day of the piglet’s life. According to Frenyo et al. [37], the highest IgG concentration was observed at 24 h after birth. Following this, a stepwise decrease occurred, and the lowest value was observed in the 4th week of age. Subsequently, there was a continuous increase until 10 weeks of age, when the concentration reached the value of that in adults.

### 4.2. Effect of Piglet Feeding on Intestinal Development and Health Status

The positive effect of MCFA on piglet performance might be due to changes in intestinal epithelium structure or in microbiota of the gastrointestinal tract; moreover, MCFA is an energy source for enterocytes that attenuates the negative effects of weaning stress on villus length and crypt depth in piglets [9]. Hanczakowska et al. [19] found a similar effect of 0.2% dietary supplement of caprylic or capric acids, wherein animals receiving caprylic acid grew faster than those receiving both these acids or capric acid alone or control ones. The present study showed very similar results: caprylic acid alone yielded better results than a mixture of MCFA (MCT supplementation), and the differences were significant especially after weaning (28–84 days of age).

Supplementation of MCFA in the diet of piglets affected their blood immunoglobulin levels. It is known that dietary fatty acids may affect immunoglobulin levels in piglet blood [38] probably due to their effects on interleukin (IL) production which, in turn, affects the production of immunoglobulin [39]. Greater serum immunoglobulin level may prevent adverse cardiovascular events [40]. Unfortunately, there are not many studies on such activity of MCFA. Nevertheless, our results are not in accordance with those of Sugiharto et al. [41], who found no effect of caproic and lauric acids on the concentration of immunoglobulin (IgA, IgM, and IgG) in piglet plasma. This difference could be due to different supplements used—caproic acid by Sugiharto et al. [41] and caprylic acid used in our experiment—and also in the composition of the basal mixture.

Small differences in blood levels of cholesterol in piglets were found by Mohana Devi and Kim [42] and by Allee et al. [43] after supplementing feed with MCFA. In an experiment on broilers, Wang et al. [44] found that total and LDL cholesterol levels in the blood of birds decreased when the content of coconut oil in feed increased. In an experiment on rats [45], MCT suppressed the levels of serum cholesterol, but similar to the present experiment, the levels of triglycerides and free fatty acids were not changed. In contrast to MCT, supplementation with caprylic acid lowered cholesterol levels in the blood of piglets. Xu et al. [46] suggested a possible mechanism through an experiment on mice. They proposed that supplementation with 2% caprylic acid in a cholesterol-rich diet reduced blood cholesterol by promoting the excretion of fecal cholesterol and cholic acid.

In the present experiment, the supplements used for feed mixtures for piglets did not affect their blood FFA level. MCT may be absorbed (intact) into the enterocytes of intestinal epithelium and then hydrolyzed in cells by microsomal lipases [47]; therefore, FFA concentration in blood may be low. Angsten et al. [48] found no increase in FFA in neonatal plasma after ingestion of MCT.

The absence of change in liver enzyme levels demonstrated the lack of toxicity of MCT [49]. The same results were noted for high doses and long feeding with MCT in clinical trials reviewed by Traul et al. [50]. Salemi and Pooya [51] showed a greater level of these enzymes in the blood of rats fed with margarine containing harmful trans-fatty acids. Alanine aminotransferase was the only enzyme whose level was lowered in the present experiment by caprylic acid supplementation. The only significant difference in liver function tests was reported by Nosaka et al. [52] in men consuming MCT or LCT; they found lower serum ALT activity in the serum of men consuming MCT than in those consuming LCT.

The feed supplements had only a small effect on the acidity of chyme in the digestive tract of piglets. MCFA had only slight effect on pH in the small intestine and showed no effect on the distal parts of the intestinal tract. This was probably due to the small doses of these acids and their rapid absorption from the proximal portions of the intestine [40]. Nowak et al. [13] noted the lack of significant influence on the cecal pH and small-chain fatty acids (SCFA) content in the gut of piglets fed with various kinds of supplements, including 0.3% MCFA; they explained this finding by the diet buffering capacity.

In monogastric animals, the large intestine is the main site for the fermentation of fibers, which are undigested in the upper portions of the intestinal tract, by bacteria that produce SCFA [53]. Thus, the lower amount of SCFA found in the cecum of piglets receiving MCT could be due to greater utilization of nutrients in the small intestine. It is also known that the amount of volatile fatty acids (VFA) in the digestive tract increases from its proximal to its distal parts. Nyachoti et al. [54] revealed that in early weaned pigs, the concentration of acetic acid changed from 0.907 mmol/L in the duodenum to 70.29 mmol/L in the ileum. In the present experiment, the concentration of VFA in the jejunum was many times lower than that in the cecum. There was a small difference in acetic acid content in the cecum of all groups; this could be because acetic acid is readily soluble in water and thus was probably absorbed in the more proximal parts of the digestive tract.

The lowering effect of MCT oil on the content of butyric and valeric acids in the cecum is difficult to explain. Both these acids are produced in situ by symbiotic bacteria [55]. The antibacterial effect of MCT is known [56], but no changes in the content of fatty acids in the jejunum were found. According to Odle [36], MCFA are absorbed from the more proximal parts of the digestive tract. A significant decrease in valeric acid content but not in butyric acid content in the cecum of piglets receiving MCFA was also found in our earlier experiment [21]. Our previous experiment [57] also showed greater digestibility of fiber when the diet of piglets was supplemented with MCFA; therefore, less substrate may be available for bacterial fermentation in the cecum. In this situation, however, the content of acetic and propionic acids should also decrease. Currently, we are unable to explain this phenomenon on the basis of the obtained results.

Very scarce data are available on the effect of fatty acids on the mass and length of piglet intestine. In our earlier experiment [21], we found no significant changes in total digestive tract weight when pure capric or caprylic acid was used. The cecum weight was lowered by caprylic acid, which is in accordance with the result of the present experiment. The only significant difference in the length of particular parts of the digestive tract was a shorter jejunum in both previous and present experiments. Furthermore, in experiments on rats and rabbits, MCT did not cause changes in organ weights [50].

Intestinal villi are the main site of nutrient absorption [58]. Their better development could be the reason for greater nutrient utilization [59], resulting in better piglet growth. Probably, the better body weight gain of piglets found in this experiment was also due to these favorable changes in the structure of the intestinal epithelium (duodenum and jejunum) as a result of MCT and especially caprylic acid consumption. Ferrara et al. [6] showed a higher extent (5%) of the positive effect on the villus height when organic acids (fumaric and lactic) were used together with caprylic and capric acids compared to organic acids alone (2.5%).

## 5. Conclusions

The type of oil supplemented in sow feed changes the fatty acid profile of milk. Supplementation of the diet with coconut oil had beneficial effects on IgM and IgG levels and lowered the mortality of piglets. Feeding piglets with caprylic acid or MCFA mixture-supplemented diets improved their performance, especially in terms of weight gain, but did not lower triacylglycerol content in blood. MCT oil significantly increased IgG level in piglet blood. Both supplements increased villus height in the duodenum, but did not change this in the jejunum. On the basis of all the results obtained, it can be concluded that coconut oil fed to pregnant and lactating sows can markedly reduce the mortality of piglets. In addition, caprylic acid and MCFA oil can be a good feed supplement in weaned piglet feed as a strategy to improve the health of piglets and thus the efficiency of pig production.

## Figures and Tables

**Table 1:** Chemical composition and fatty acid composition of basal feeds supplemented with different oils, served for sows during late pregnancy and lactation.

Item	Mixtures Containing Rapeseed Oil (RO)	Mixtures Containing Coconut Oil (CO)
*Ingredients (g/kg)*		
Corn meal	240	240
Wheat, ground	150	150
Barley, ground	100	100
Triticale	100	100
Wheat bran	150	150
Soybean meal	150	150
Dried grass	50	50
Rapeseed oil	20	-
Coconut oil	-	20
LK Global Max ^1^	40	40
*Analyzed composition (g/kg)*		
Dry matter	892.1	893.2
Crude protein (N × 6.25)	167.9	167.0
Ether extract	23.4	35.6
Crude fiber	50.6	52.0
Crude ash	58.9	58.6
Metabolizable energy (MJ/kg) ^2^	12.6	12.6
*Fatty acids composition (% of total fatty acids)*		
C6:0	0.43	0.75
C8:0	0.01	0.60
C10:0	0.03	2.31
C12:0	0.10	16.78
C14:0	0.13	5.42
C16:0	10.18	11.68
C16:1	0.06	0.03
C18:0	1.49	1.44
C18:1	37.98	15.64
C18:2	41.75	40.40
C18:3	7.21	4.28
C20:4	0.29	0.21
C22:1	0.21	0.06

^1^ LK Global Max (Cargill Ltd., Kiszkowo, Poland) provided the following per kilogram of complete diet: Metabolic Energy, 2.6 MJ; crude protein, 100 g; Lys, 60 g; Met + Cys, 12 g; Thr, 21 g; Val, 10 g; Ca, 210 g; P digestible, 70 g; Na, 50 g; vitamin A, 380,000 IU; vitamin D, 50, 000 IU; vitamin E, 3750 mg; vitamin K, 100 mg; vitamin B1, 57 mg; vitamin B2, 152 mg; vitamin B6, 114 mg; vitamin B12, 950 µg; vitamin C, 2500 mg; folic acid, 125 mg; pantothenic acid, 400 mg; nicotinic acid, 760 mg; biotin, 7500 µg; choline cloride, 8625 mg; Mn, 1330 mg; Zn, 3010 mg; Fe, 3000 mg; Cu, 510 mg; I, 34 mg; Se,11,1 mg, and antioxidant, phytase, and trace elements, combinations of organic; ^2^ Metabolizable energy was calculated using equation from [23].

**Table 2:** Components and chemical composition of piglet diets.

Item	7 to 21 Days of Age			22 to 84 Days of Age		
	NS	MCT	C8	NS	MCT	C8
*Ingredient (g/kg)*						
Wheat, ground	450	450	450	300	300	300
Barley, ground	179	177	177	341	339	339
Soybean meal	120	120	120	220	220	220
Soybean meal (HP300) ^3^	100	100	100	-	-	-
Skim milk powder	60	60	60	50	50	50
Dried whey	60	60	60	60	60	60
Calcium phosphate	10	10	10	7	7	7
Calcium carbonate	11	11	11	11	11	11
Premix ^1, 2^	5	5	5	5	5	5
Salt	3	3	3	3	3	3
L-lysine	2	2	2	3	3	3
MCT oil ^4^	-	3	-	-	3	-
Caprylic acid (C8:0)	-	-	3	-	-	3
*Composition in 1 kg of feed mixture (g/kg)*						
Dry matter	892.4	892.4	892.4	879.3	879.3	879.3
Crude protein	210	210	210	202	202	202
Ether extract	11.4	11.4	11.4	8.1	8.1	8.1
Crude fiber	28.3	28.3	28.3	27.0	27.0	27.0
Crude ash	57.9	57.9	57.9	56.7	56.7	56.7
Metabolizable energy (MJ/kg) ^5^	13.3	13.3	13.3	13.2	13.2	13.2

NS, no supplement; MCT, MCT oil; C8, acid C8:0; ^1^ Premix used 7 to 21 d of age provided the following per kilogram of complete diet: VA, 15,000 IU; VD3, 1,500 IU; VE, 105 mg; VK3, 2.25 mg; VB1, 2.25 mg; VB2, 6 mg; VB6, 4.5 mg; VB12, 0.04 mg; pantothenic acid, 15 mg; choline chloride, 400 mg; folic acid, 2 mg; nicotinic acid, 20 mg; Mg, 50 mg; Mn, 40 mg; I, 0.8 mg; Zn, 140 mg; Fe, 100 mg; Cu, 160 mg; Co, 0.4 mg; Se, 0.2 mg; complete limestone to 5000 mg; ^2^ Premix used 21 to 70 d of age provided the following per kilogram of complete diet: VA, 12,000 IU; VD3, 1500 IU; VE, 70 mg; VK3, 1.5 mg; VB1, 1.5 mg; VB2, 6 mg; VB6, 4.5 mg; VB12, 0.025 mg; pantothenic acid, 10 mg; choline chloride, 400 mg; folic acid, 2 mg; nicotinic acid, 20 mg; Mg, 50 mg; Mn, 40 mg; I, 0.8 mg; Zn, 140 mg; Fe, 100 mg; Cu, 160 mg; Co, 0.4 mg; Se, 0.2 mg; complete limestone to 5000 mg; ^3^ Hydrolyzed soy protein HP 300, Hamlet Protein A/S, Horsens, Denmark; ^4^ MCT oil, Medium Chain Triglycerides, Olimp Laboratories Sp. z o.o., Dębica, Poland; ^5^ Metabolisable energy was calculated using equation from [23].

**Table 3:** Sows reproductive rates.

Item	RO	CO	s.e.m.	*p*-Value
Number of sows (No.)	12	12	-	-
Sow body weight at 100th day of gestation (kg)	263	252	7.14	0.45
Sow body weight at farrowing (kg)	249	237	6.96	0.43
Body weight change (d 100 to farrowing) (kg)	−14.4	−14.6	1.65	0.96
Sow body weight at weaning (kg)	211	202	6.70	0.53
Body weight loss during lactation (kg)	37.7	35.0	3.50	0.71
Number of piglets born per litter (No.)	11.8	11.7	0.41	0.23
Body weight of piglets at 1 d of age (kg)	1.78	1.77	0.02	0.91
Body weight of piglets at 7 d of age (kg)	3.12	3.24	0.05	0.45
Average daily weight gain 1 to 7 d (g)	223	245	6.26	0.27
Piglet losses 1 to 7 d (No.)	8	2	-	-
Piglet losses 1 to 7 d (%)	5.7	1.4	-	-

RO, rapeseed oil; CO, coconut oil; s.e.m., standard error of the mean.

**Table 4:** Fatty acids composition (% of total fatty acids) of sows milk fed with different oils.

Fatty Acid	RO	CO	s.e.m.	*p*-Value
C6:0	0.18	0.17	0.01	0.66
C8:0	0.07	0.06	0.01	0.57
C10:0	0.44	0.42	0.03	0.71
C12:0	0.80 ^a^	3.52 ^b^	0.47	<0.01
C14:0	5.96 ^a^	7.77 ^b^	0.38	<0.01
C16:0	38.63	35.83	0.97	0.16
C16:1	3.51	3.56	0.19	0.90
C18:0	3.66	3.68	0.18	0.96
C18:1	33.72	34.50	1.25	0.77
C18:2	10.70 ^b^	9.01 ^a^	0.33	<0.01
C18.3 n3	0.09	0.09	0.01	0.86
C18:3 n6	1.60 ^b^	0.92 ^a^	0.11	<0.01
C20:0	0.013 ^b^	0.003 ^a^	0.002	<0.01
CLA t9–t11	0.02 ^b^	0.01 ^a^	0.00	0.02
C20:4	0.44	0.36	0.03	0.09
C22:1	0.08 ^b^	0.04 ^a^	0.01	0.04
EPA	0.070 ^b^	0.036 ^a^	0.01	0.04
DHA	0.013 ^b^	0.003 ^a^	0.002	0.02
SFA	49.76	51.46	1.24	0.52
UFA	50.24	48.54	1.24	0.52
MUFA	37.31	38.11	1.11	0.74
PUFA	12.94 ^b^	10.44 ^a^	0.45	<0.01
PUFA n6	11.23 ^b^	9.47 ^a^	0.35	<0.01
PUFA n3	1.69 ^b^	0.96 ^a^	1.12	<0.01
PUFA n6/n3	6.76 ^b^	9.89 ^a^	0.53	<0.01

RO, rapeseed oil; CO, coconut oil; EPA, eicosapentaenoic acid; DHA, docosahexaenoic acid; SFA, sum of saturated fatty acids; UFA, total sum of unsaturated fatty acids; MUFA, sum of monounsaturated fatty acids; PUFA, sum of polyunsaturated fatty acids; ^a,b^ Values within a row with different superscripts were significantly different (*p* < 0.05). s.e.m., standard error of the mean.

**Table 5:** Indices of piglet performance.

Factors		Number Piglets Born (No)	Dead and Culled Piglets (%)	Number Piglets (No)	Body Weight on Day of Age (kg)				Average Body Weight Gains of Piglets (g)				
ToO	SM				1 Day	28 Days	56 Days	84 Days	1–28 Days	28–56 Days	56–84 Days	28–84 Days	1–84 Days
*Treatment*													
RO	NS	48	16.7	40	1.70	7.09 ^a^	11.28 ^a^	24.26 ^a^	200 ^a^	150 ^a^	464 ^a^	307 ^a^	272 ^a^
	MCT	43	4.7	41	1.85	7.64 ^a,b^	12.46 ^a,b^	26.07 ^a,b,c^	214 ^a,b^	172 ^a,b^	486 ^a,b^	329 ^a^	292 ^a,b,c^
	C8	50	8.0	46	1.79	8.42 ^b^	14.35 ^c^	27.93 ^b,c^	246 ^b^	212 ^c^	485 ^a,b^	348 ^a,b^	315 ^b,c^
CO	NS	52	7.7	52	1.74	7.69 ^a,b^	12.39 ^a,b^	25.42 ^a,b^	220 ^a,b^	168 ^a^	465 ^a^	316 ^a^	285 ^a,b^
	MCT	47	0	45	1.76	8.37 ^b^	12.90 ^b,c^	27.51 ^b,c^	245 ^b^	162 ^a^	522 ^a,b^	342 ^a,b^	310 ^b,c^
	C8	41	0	39	1.80	7.48 ^a,b^	13.20 ^b,c^	28.86 ^c^	210 ^a^	205 ^b^	559 ^b^	382 ^b^	326 ^c^
s.e.m.					0.022	0.104	0.169	0.309	3.488	3.909	3.648	7.792	4.849
*Main factors*													
RO		141	9.9	127	1.78	7.71	12.69	26.09 ^a^	217	178	478 ^a^	328 ^a^	292 ^a^
CO		140	2.9	136	1.77	7.85	12.83	27.27 ^b^	224	178	515 ^b^	347 ^b^	307 ^b^
	NS	100	12.0	88	1.72	7.39 ^a^	11.83 ^a^	24.84 ^a^	210 ^a^	159 ^a^	464 ^a^	311 ^a^	278 ^a^
	MCT	90	2.2	88	1.80	8.00 ^b^	12.68 ^b^	26.79 ^b^	230 ^b^	167 ^a^	504 ^a,b^	336 ^a^	301 ^b^
	C8	91	4.4	87	1.80	7.95 ^a,b^	13.77 ^b^	28.40 ^b^	228 ^b^	208 ^b^	522 ^b^	365 ^b^	320 ^b^
*p*-value													
ToO					0.769	0.524	0.672	0.048	0.437	0.972	0.016	0.045	0.042
SM					0.175	0.021	<0.001	<0.001	0.030	<0.001	0.006	<0.001	<0.001
ToO × SM					0.399	0.001	0.015	0.944	<0.001	0.211	0.145	0.539	0.913

ToO, type of oil: RO, rapeseed oil; CO, coconut oil; SM, supplemental medium: NS, no supplement; MCT, MCT oil; C8, acid C8:0; ^a,b,c^ Values within a column with different superscripts were significantly different (*p* < 0.05); s.e.m., Standard error of the mean.

**Table 6:** Acidity of chyme in the stomach and in various parts of intestines in piglets at 60th day of age.

Factors		Stomach	Duodenum	Jejunum	Cecum	Colon
ToO	SM					
*Treatment*						
RO	NS	3.24	5.76 ^b,c^	5.81	5.38 ^a,b^	5.61
	MCT	3.47	5.72 ^c^	5.95	5.60 ^b^	5.87
	C8	3.14	5.70 ^c^	5.82	4.96 ^a^	5.14
CO	NS	3.14	5.88 ^c^	5.85	5.13 ^a^	5.74
	MCT	3.26	5.33 ^a,b^	5.76	5.13 ^a^	5.65
	C8	3.24	5.00 ^a^	5.79	5.34 ^a,b^	5.74
s.e.m.		0.07	0.07	0.05	0.05	0.08
*Main factors*						
RO		3.28	5.73	5.86	5.31	5.54
CO		3.21	5.40	5.80	5.20	5.71
	NS	3.19	5.82 ^c^	5.83	5.26	5.68
	MCT	3.36	5.52 ^b^	5.86	5.36	5.76
	C8	3.19	5.35 ^a^	5.80	5.15	5.44
*p*-value						
ToO		0.63	<0.01	0.57	0.19	0.29
SM		0.53	<0.01	0.92	0.14	0.23
ToO × SM		0.66	<0.01	0.68	<0.01	0.12

Number of observations included in the calculation of means for treatment effects are 6, whereas for means for main effects are 18 (type of oil) or 12 (supplemental medium); ToO, type of oil: RO, rapeseed oil; CO, coconut oil; SM, supplemental medium: NS, no supplement; MCT, MCT oil; C8, acid C8:0; ^a,b,c^ Values within a column with different superscripts were significantly different (*p* < 0.05); s.e.m., Standard error of the mean.

**Table 7:** Various parts of intestines and volatile fatty acid (VFA) content of piglets’ chime (μmol/g chyme) at 60th day of age.

Factors		Jejunum							Cecum						
ToO	SM	Acetic	Propionic	Isobutyric	Butyric	Isovaleric	Valeric	Total	Acetic	Propionic	Isobutyric	Butyric	Isovaleric	Valeric	Total
*Treatment*															
RO	NS	7.29	0.29	0.04	0.32	0.04 ^a^	0.04	8.02	79.92 ^b^	43.88 ^a,b^	1.43	26.96 ^b^	1.12	6.28 ^a,b^	159.60 ^b^
	MCT	8.26	0.21	0.03	0.22	0.07 ^a,b^	0.05	8.85	59.93 ^a^	37.85 ^a^	1.09	15.52 ^a^	0.51	2.74 ^a^	117.65 ^a^
	C8	5.72	0.27	0.03	0.31	0.07 ^a,b^	0.05	6.46	69.02 ^a,b^	45.68 ^a,b^	1.72	26.71 ^a,b^	0.97	6.54 ^a,b^	150.64 ^a,b^
CO	NS	6.29	0.24	0.05	0.26	0.05 ^a,b^	0.05	6.94	74.31 ^a,b^	54.35 ^b^	2.08	27.75 ^a,b^	0.55	7.40 ^b^	166.43 ^b^
	MCT	6.40	0.33	0.03	0.25	0.10 ^b^	0.07	7.18	82.26 ^b^	49.33 ^a,b^	2.37	18.72 ^a,b^	0.92	3.59 ^a,b^	157.19 ^b^
	C8	7.02	0.40	0.05	0.33	0.08 ^a,b^	0.05	7.93	72.50 ^a,b^	41.21 ^a^	1.46	21.71 ^a,b^	0.70	4.59 ^a,b^	142.17 ^a,b^
s.e.m		0.29	0.04	0.01	0.02	0.01	0.01	0.32	2.22	1.30	0.14	1.24	0.09	0.44	4.18
*Main factors*															
RO		7.09	0.26	0.04	0.28	0.06	0.05	7.78	69.62	42.47	1.42	23.07	0.087	5.18	142.63
CO		6.57	0.33	0.04	0.28	0.08	0.05	7.35	76.36	48.30	1.97	22.07	0.72	5.19	155.27
	NS	6.79	0.27	0.04	0.29	0.05 ^a^	0.05	7.48	77.12	49.11	1.76	27.36 ^b^	0.85	6.84 ^b^	163.01 ^b^
	MCT	7.33	0.27	0.03	0.23	0.09 ^b^	0.06	8.02	71.10	43.59	1.73	17.12 ^a^	0.71	3.16 ^a^	137.42 ^a^
	C8	6.37	0.34	0.04	0.32	0.07 ^a,b^	0.05	7.20	70.76	43.44	1.59	24.21 ^b^	0.84	5.56 ^b^	146.41 ^a,b^
*p*-value															
ToO		0.37	0.34	0.34	0.92	0.22	0.33	0.50	0.11	0.01	0.04	0.88	0.42	0.99	0.09
SM		0.39	0.65	0.60	0.32	0.02	0.08	0.55	0.38	0.08	0.96	<0.01	0.81	<0.01	0.03
ToO × SM		0.08	0.54	0.74	0.73	0.68	0.70	0.10	0.03	<0.01	0.06	0.29	0.09	0.21	0.03

Number of observations included in the calculation of means for treatment effects are 6, whereas for means for main effects are 18 (type of oil) or 12 (supplemental medium); ToO, type of oil: RO, rapeseed oil; CO, coconut oil; SM, supplemental medium: NS, no supplement; MCT, MCT oil; C8, acid C8:0; ^a,b^ Values within a column with different superscripts were significantly different (*p* < 0.05); s.e.m., Standard error of the mean.

**Table 8:** Mass and length of piglet intestines and mass of internal organs at 60th day of age.

Factors		Intestinal Mass/BW (g/kg)					Intestinal Length/BW (cm/kg)					Mass of Organs/BW (g/kg)				
ToO	SM	Duodenum	Jejunum	Cecum	Colon	Total Dig. Tract	Duodenum	Jejunum	Cecum	Colon	Total Dig. Tract	Liver	Heart	Kidney	Spleen	Internal Fat
*Treatment*																
RO	NS	0.85 ^a^	40.03	2.63 ^b^	13.87 ^a,b^	57.39 ^a,b^	1.52	76.43 ^b^	1.06 ^b^	15.15 ^b^	94.17 ^b^	23.31 ^a^	5.04	2.75	1.37 ^a^	1.94
	MCT	1.16 ^a,b^	44.91	2.52 ^a,b^	15.65 ^b^	64.25 ^b^	1.59	66.75 ^a,b^	1.00 ^a,b^	14.95 ^b^	84.29 ^a,b^	28.78 ^b^	6.30	3.04	2.15 ^b^	2.40
	C8	1.00 ^a,b^	43.65	2.22 ^a,b^	14.81 ^a,b^	61.68 ^a,b^	1.29	69.68 ^a,b^	0.81 ^a^	14.02 ^a,b^	85.81 ^a,b^	25.63 ^a,b^	5.78	2.65	1.70 ^a^	2.07
CO	NS	1.04 ^a,b^	4273	2.29 ^a,b^	15.98 ^b^	62.04 ^a,b^	1.51	74.91 ^a,b^	1.01 ^a,b^	15.31 ^b^	92.74 ^b^	24.76 ^a,b^	5.30	2.63	1.68 ^a^	1.80
	MCT	1.37 ^b^	46.31	3.35 ^c^	14.02 ^a,b^	65.05 ^b^	1.60	81.73 ^b^	1.15 ^b^	13.58 ^a,b^	98.06 ^b^	24.52 ^a^	5.97	2.46	1.78 ^a,b^	1.53
	C8	0.81 ^a^	38.67	2.00 ^a^	12.46 ^a^	53.94 ^a^	1.24	57.72 ^a^	0.82 ^a^	11.63 ^a^	71.40 ^a^	24.97 ^a,b^	5.41	2.44	1.72 ^a^	1.96
s.e.m		0.05	0.87	0.09	0.32	1.11	0.04	2.02	0.03	0.35	2.28	0.45	0.22	0.08	0.05	0.12
*Main factors*																
RO		1.00	42.86	2.46	14.78	61.10	1.47	70.95	0.96	13.81	88.09	25.91	5.71	2.81	1.74	2.75
CO		1.07	42.57	2.55	14.16	60.34	1.45	71.45	0.99	13.51	87.40	24.75	5.56	2.51	1.73	1.76
	NS	0.95 ^a,b^	41.38	2.47 ^b^	14.93	57.71 ^a,b^	1.52 ^b^	75.67 ^b^	1.03 ^b^	15.23 ^b^	93.45 ^b^	24.03 ^a^	5.17	2.69	1.52 ^a^	2.79
	MCT	1.27 ^b^	45.61	2.94 ^c^	14.84	64.65 ^b^	1.60 ^b^	74.24 ^b^	1.07 ^b^	14.27 ^a,b^	91.18 ^b^	26.65 ^b^	6.13	2.75	1.96 ^b^	1.96
	C8	0.90 ^a^	41.16	2.11 ^a^	13.63	57.81 ^a^	1.26 ^a^	63.70 ^a^	0.82 ^a^	11.47 ^a^	78.60 ^a^	25.30 ^a,b^	5.60	2.54	1.71 ^a,b^	2.01
*p*-value																
ToO		0.45	0.86	0.44	0.26	0.70	0.82	0.88	0.39	0.75	0.85	0.14	0.75	0.06	0.89	0.18
SM		<0.01	0.06	<0.01	0.10	0.02	<0.01	0.01	<0.01	<0.01	<0.01	0.03	0.24	0.52	<0.01	0.59
ToO × SM		0.12	0.12	<0.01	<0.01	0.03	0.95	<0.01	0.12	0.73	0.02	0.02	0.82	0.42	<0.01	0.57

Number of observations included in the calculation of means for treatment effects are 6, whereas for means for main effects are 18 (type of oil) or 12 (supplemental medium); ToO, type of oil: RO, rapeseed oil; CO, coconut oil; SM, supplemental medium: NS, no supplement; MCT, MCT oil; C8, acid C8:0; ^a,b,c^ Values within a column with different superscripts were significantly different (*p* < 0.05); s.e.m., Standard error of the mean.

**Table 9:** Mucosal epithelium structure of the duodenum and jejunum (μm) of piglets at 60th day of age.

Factors		Duodenum				Jejunum			
ToO	SM	Villus Height	Villus Width	Crypt Depth	Villus Height/Crypt Depth	Villus Height	Villus Width	Crypt Depth	Villus Height/Crypt Depth
*Treatment*									
RO	NS	361	169	355 ^a^	1.04 ^a,b^	333	137 ^a^	302	1.10
	MCT	393	179	384 ^a,b^	1.03 ^a b^	343	154 ^a,b^	339	1.01
	C8	418	181	351 ^a,b^	1.19 ^b^	340	132 ^a^	307	1.12
CO	NS	332	152	390 ^a,b^	0.85 ^a^	327	139 ^a,b^	341	0.96
	MCT	348	164	362 ^a,b^	0.96 ^a^	342	167 ^b^	308	1.12
	C8	457	220	482 ^b^	0.94 ^a^	402	154 ^a,b^	351	1.14
s.e.m		14.98	7.29	14.04	0.03	9.14	3.00	5.97	0.02
*Main factors*									
RO		391	176	363	1.09	339	141	316	1.08
CO		379	179	411	0.92	357	153	333	1.07
	NS	347 ^a^	160	373	0.94	330	138 ^a^	320	1.03
	MCT	370 ^a,b^	172	373	0.99	343	160 ^b^	324	1.07
	C8	438 ^b^	200	417	1.07	371	143 ^a^	329	1.13
*p*-value									
ToO		0.69	0.86	0.07	<0.01	0.32	0.02	0.14	0.92
SM		0.04	0.07	0.30	0.07	0.18	<0.01	0.08	0.23
ToO × SM		0.46	0.19	0.07	0.21	0.24	0.29	0.02	0.10

Number of observations included in the calculation of means for treatment effects are 6, whereas for means for main effects are 18 (type of oil) or 12 (supplemental medium); ToO, type of oil: RO, rapeseed oil; CO, coconut oil; SM, supplemental medium: NS, no supplement; MCT, MCT oil; C8, acid C8:0; ^a,b,c^ Values within a column with different superscripts were significantly different (*p* < 0.05); s.e.m., Standard error of the mean.

**Table 10:** Biochemical indices in serum of piglet at 60th day of age.

Factors		Cholesterol				TG (mmol/L)	FFA (mmol/L)	ALP (U/L)	ALT (U/L)	AST (U/L)	LDH (U/L)
ToO	SM	Total (mmol/L)	LDL (mmol/L)	HDL (mmol/L)	HDL (%)						
*Treatment*											
RO	NS	1.48 ^b^	0.57 ^b^	0.68 ^a,b,c^	46.2 ^a,b^	0.50 ^b^	0.23 ^b^	200.9 ^a^	75.2 ^b^	32.9 ^a,b^	1505
	MCT	1.59 ^b^	0.69 ^b,c^	0.67 ^a,b^	42.0 ^a^	0.51 ^b^	0.19 ^b^	233.8 ^b^	72.6 ^a,b^	30.5 ^a^	1577
	C8	1.16 ^a^	0.37 ^a^	0.59 ^a^	50.9 ^c^	0.45 ^a,b^	0.18 ^b^	220.1 ^a,b^	62.2 ^a^	41.2 ^b^	1658
CO	NS	1.76 ^c^	0.79 ^c^	0.79 ^c^	44.8 ^a,b^	0.41 ^a,b^	0.11 ^a^	220.1 ^a,b^	78.1 ^b^	30.1 ^a^	1579
	MCT	1.60 ^b^	0.66 ^b,c^	0.77 ^b,c^	48.2 ^b,c^	0.38 ^a^	0.09 ^a^	211.1 ^a,b^	71.0 ^a,b^	25.8 ^a^	1432
	C8	1.53 ^b^	0.61 ^b^	0.70 ^b,c^	46.0 ^a,b^	0.47 ^a,b^	0.10 ^a^	193.5 ^a^	61.3 ^a^	24.0 ^a^	1539
s.e.m. ^5^		0.04	0.03	0.02	0.6	0.01	0.01	3.4	1.5	1.2	28.1
*Main factors*											
RO		1.41	0.51	0.65	46.4	0.49	0.20	218.3	70.0	34.9	1580
CO		1.63	0.69	0.75	46.3	0.42	0.10	208.2	70.1	26.7	1516
	NS	1.61 ^b^	0.68 ^b^	0.74 ^b^	45.5 ^a^	0.46	0.17	210.5	76.7 ^b^	31.5	1542
	MCT	1.59 ^b^	0.68 ^b^	0.72 ^b^	45.1 ^a^	0.44	0.14	222.4	71.8 ^b^	28.2	1504
	C8	1.34 ^a^	0.49 ^a^	0.65 ^a^	48.4 ^b^	0.46	0.14	206.8	61.8 ^a^	32.6	1599
*p*-value											
ToO		<0.01	<0.01	<0.01	0.94	<0.01	<0.01	0.08	0.95	<0.01	0.26
SM		<0.01	<0.01	<0.01	<0.01	0.83	0.07	0.07	<0.01	0.13	0.38
ToO × SM		<0.01	<0.01	0.99	<0.01	0.03	0.36	<0.01	<0.01	0.13	0.23

Number of observations included in the calculation of means for treatment effects are 6, whereas for means for main effects are 18 (type of oil) or 12 (supplemental medium); ToO, type of oil: RO, rapeseed oil; CO, coconut oil; SM, supplemental medium: NS, no supplement; MCT, MCT oil; C8, acid C8:0; ^a,b,c^ Values within a column with different superscripts were significantly different (*p* < 0.05); s.e.m., Standard error of the mean.

**Table 11:** Immunoglobin content (mg/dL) and C-reactive protein (mg/L) in piglet serum.

Factors		21 Days of Age				60 Days of Age			
ToO	SM	IgM	IgA	IgG	CRP	IgM	IgA	IgG	CRP
*Treatment*									
RO	NS	31.0 ^a,b^	4.6	224.8 ^a,b^	2.21	64.6	7.3	287.8 ^a,b^	1.82
	MCT	23.9 ^a^	6.3	257.0 ^b^	1.23	66.7	8.6	320.3 ^a,b^	1.78
	C8	24.2 ^a^	3.2	147.8 ^a^	2.14	67.7	4.3	251.0 ^a^	1.97
CO	NS	36.0 ^a,b^	5.6	292.6 ^b^	1.86	65.5	5.1	256.8 ^a^	1.85
	MCT	38.6 ^b^	5.0	222.2 ^a,b^	2.38	71.6	6.8	355.5 ^b^	2.19
	C8	27.0 ^a,b^	4.0	272.4 ^b^	1.64	55.2	5.7	302.3 ^a,b^	1.67
s.e.m. ^5^		1.4	0.3	10.2	0.16	2.1	0.5	8.9	0.13
*Main factors*									
RO		26.4	4.7	209.9	1.86	66.3	6.8	286.4	1.92
CO		33.9	5.8	262.4	1.96	64.1	5.9	303.2	1.93
	NS	33.5 ^b^	5.1 ^a,b^	258.7 ^b^	2.03	65.0	6.2 ^a,b^	272.3 ^a^	1.89
	MCT	31.3 ^a,b^	5.7 ^b^	239.6 ^a,b^	1.81	69.2	7.7 ^b^	335.4 ^b^	1.88
	C8	25.6 ^a^	3.6 ^a^	210.1 ^a^	1.90	61.4	5.0 ^a^	276.7 ^a^	2.00
*P*-value									
ToO		<0.01	0.86	<0.01	0.74	0.61	0.33	0.27	0.99
SM		0.03	0.03	0.04	0.82	0.30	0.05	<0.01	0.93
ToO × SM		0.11	0.26	<0.01	0.07	0.20	0.18	0.09	0.24

Number of observations included in the calculation of means for treatment effects are 6, whereas for means for main effects are 18 (type of oil) or 12 (supplemental medium); ToO, type of oil: RO, rapeseed oil; CO, coconut oil; SM, supplemental medium: NS, no supplement; MCT, MCT oil; C8, acid C8:0; ^a,b^ Values within a column with different superscripts were significantly different (*p* < 0.05); s.e.m., Standard error of the mean.
